# The use of Web-based interactive technology to promote HPV vaccine uptake among young females: a randomized controlled trial

**DOI:** 10.1186/s12905-021-01417-y

**Published:** 2021-07-30

**Authors:** Qi Wang, Wen Zhang

**Affiliations:** 1 School of Industrial Design, Hubei University of Technology, 28 Nanli Road, Wuhan, 430068 People’s Republic of China; 2 School of Journalism and Culture Communication, Zhongnan University of Economics and Law, 182 Nanhu Avenue, Wuhan, 430073 People’s Republic of China

**Keywords:** Human papillomavirus, Web-based interactivity, Narratives, Data visualization, Information avoidance, HPV vaccination intention

## Abstract

**Background:**

Currently no study has investigated whether Web-based interactive technology can influence females to adopt healthy behaviors. We investigated how and under what conditions do Web-based interactivity influence vaccination intentions among young females.

**Methods:**

In this randomized controlled trail, we conduct a 2 (mode of information presentation: narrative vs. data visualization) × 2 (interactivity: interactive information vs. noninteractive information) between-groups design. A total of 180 Chinese female undergraduate students who had never received HPV vaccination were randomly allocated to 4 experimental groups. Each participant was assessed for their information avoidance behavior and vaccination intention. The hypotheses were tested using a moderated mediation model. All analyses were performed using SPSS version 22.0 with probability set at 0.05 alpha level.

**Results:**

The indirect relationship between interactivity and behavioral intention though information avoidance was moderated by the mode of presentation. Under the narrative condition, interactivity (vs. non-interactivity) decreased information avoidance and increased the intention to receive HPV vaccination (B = -.23, SE = 0.10, P < 0.05). However, under data visualization condition, no significant difference was observed between the effects of interactivity and non-interactivity on intention.

**Conclusion:**

The findings suggest that when young females experience difficulties in manipulating or understanding HPV-related information, their information-avoidance behavior is likely to increase. Rather than use interactive statistical or graphical information, young females are more likely to be persuaded by interactive narratives.

## Background

Persistent human papillomavirus (HPV) infection has been associated with the development of malignancies such as cervical and oropharyngeal cancers [1]. At the beginning of 2020, a relativity affordable (about US$140) domestic bivalent HPV vaccine (Cecolin) was approved and put on the market in mainland China. However, a meta-analysis of 53 articles with 82,813 respondents showed that only 17.13% of young women are aware of the HPV vaccine and nearly half have not accepted to be vaccinated [2]. The primary reasons for not accepting the vaccine included concerns of side effects, lack of sufficient knowledge, low awareness [3] and moral obligation and STD-related stigma [4]. Interventions that increase knowledge about HPV, a common virus that cause six types of cancer, and HPV vaccine are among the key approaches used to promote HPV vaccination in China.

Since women are more likely to search for health-related information online or ask family members or friends [5], they are a target group likely to benefit from healthcare information offered online. Of note, women are less receptive of information concerning sexually transmitted diseases such as HIV/AIDS or HPV publicly in fear of being ostracized or stigmatized [6]. Such women feel shielded from embarrassment and being judged when accessing health information online since it is a private and anonymous platform [7]. Against this background, governments and mass media companies now provide information related to HPV vaccination targeting women through eHealth programs with aim of encouraging them to accept HPV vaccination [8].

With the rapid development of smartphones, multimedia and the internet tools, web-based interactive technology is considered an important user-driven tool through which health scientific information can be understood [9]. Users can interact with the information by clicking, dragging, and zooming, hence they can get closer to the information almost anytime and anyplace [10]. Interactive technology enable users to access large amount of information based on their natural perceptual abilities [11]. For example, Pedigree Visualization and Navigation (PViN), an interactive information tool, helps practitioners and patients better to understand the risks associated with hereditary diseases [11]. Similarly, the web-based interactive software Medhisory allows efficient viewing of medication information [12].

Moreover, web-based interactivity among females is critical for promotion of HPV vaccination since women or girls are the primary target for HPV vaccination. Research from a techno-anxiety perspective has shown that individuals may be hesitant, fearful, or simply unwilling to use new technology [13]. Some researchers have argued that women are more likely than men to report higher techno- or computer anxiety [14–16] and generally avoid manipulating products that require an element of skill [17]. As a result, investigations should be conducted to determine how gender impacts the design of interactive health information and its application.

Although there is ample evidence that eHealth motivates women to search for healthcare information [7, 18], few studies have investigated the persuasiveness of Web-based interactive information on women. To close this gap, we combined the theory of information avoidance [19] and the limited capacity model of motivated mediated messages (LC4MP) developed by Lang [20] to investigate the design and persuasive effect of Web-based interactive health information on women. The specific aims of this study followed the techno-anxiety perspective. They include: [1] conduct a between-groups experimental study on 180 unvaccinated (HPV vaccine) Chinese females using/not using Web-based interactive technology; [2] explore the acceptability of Web-based interactive messages related to cancer among unvaccinated young females; [3] investigate the interplay between interactivity and the presentation of information to extend our understanding of the nature of interaction with interactive features and message content, and how it induces information avoidance behavior and further influences vaccination intention.

## Literature review

### Web-based interactive technology and health behavioral intention

Interactivity refers to the “technological attributes of mediated environments that enable reciprocal communication or information exchange, which afford interaction between communication technology and users, or between users through technology” [21]. Specifically, the current study defines interactivity as accessing Web-based information using a mouse or touchscreen through pointing, clicking, scrolling, dragging or flipping to manipulate the content [22].

Interacting with information is expected to shape more positive perceptions and attitudes, which may enhance users’ behavioral intentions to follow the advice provided [23, 24]. Lu et al. [25] modelled four websites designed to promote physical activity among college students and found that Web interactivity affected participants’ intentions to visit a fitness center. O’Leary et al. [26] examined the impact of Web-based interactivity on the uptake of maternal vaccines and demonstrated that the website with vaccine information and interactive social media components positively influenced vaccine uptake.

Although Web-based interactive technology seems to increase the persuasiveness of health messages, previous studies on interactivity have focused primarily on interactions among people and Web2.0 technology such as sharing, tagging, and instant messaging [27], or on-screen interaction techniques for promoting goods on e-commerce sites [28]. There is insufficient evidence that the typical type of interactivity interfaced with health content can enhance awareness of HPV and behavioral intentions to obtain the vaccine.

### Web-based interactivity, LC4MP and information avoidance

Information overload motivates information avoidance [29]. Information avoidance refers to behavior intended to avoid the acquisition of available information [30]. Individuals are more likely to avoid useful information if it causes them to feel anxious [31]. Studies from a psychosocial perspective have indicated that individuals may eschew information to avoid distress [32], prevent a change in belief [30], or delay an undesired action [33]. On the other hand, studies based on LC4MP suggest that people have a limited capacity to engage in the tasks of encoding, storage, and retrieval of information. If a task requires a high level of encoding that dominates cognitive resources, it leaves insufficient resources available for information storage and retrieval [20, 28]. Information avoidance occurs when people encounter complex or incomprehensible information that overwhelms their limited information processing capacities [34]. Interactive information is more complex to manipulate than static information and may compete for limited cognitive resources and trigger cognitive overload. Thus, we propose:

#### **Hypothesis 1**

Interactivity in the delivery of HPV vaccine-related information will increase information avoidance.

Information avoidance is detrimental to public health because it is negatively related to health knowledge and the adoption of preventive behaviors [35]. Pomares et al. [36] examined the link between cognitive processes and HPV vaccine hesitancy among parents of adolescents and found that information avoidance was associated with vaccine hesitancy. We therefore propose:

#### **Hypothesis 2**

Information avoidance influences the behavioral intention to obtain the HPV vaccine.

#### **Hypothesis 3**

Information avoidance mediates the effect of interactivity on the intention to receive the HPV vaccine.

### Narratives and data visualization

Narratives and statistics are the two most common ways to present information about HPV vaccination to the public. Narratives, in the form of storytelling, increase non-experts’ comprehension of, interest in, and engagement with HPV information [37]. Conversely, the government and NGOs tend to emphasize the risks of HPV and the importance of vaccination using statistics and probabilities, and data visualization is often used to present quantitative data [38]. However, a significant proportion of the general public has low graphic literacy. Narrative information is often more powerful than statistical information [39–41].

Interactive narratives and interactive data visualization are processed in qualitatively different ways. Interactive narratives allow users to control the direction of the story’s plot. They are seen as a useful tool to increase the audience’s knowledge about a health issue and change health behavior [42]. Users enact and drive the narrative rather than merely witnessing the story, so it is more immersive and entertaining than a traditional program. Although interactive narratives require some familiarity with technology, the interactive element does not make them more difficult to understand than traditional narratives [43]. In contrast, interactive data visualization is basically descriptive and collective, and thus requires more cognitive resources to understand than an interactive narrative. Interactive features such as scrolling, zooming, and dragging are more complicated to manipulate. Therefore, information avoidance may be less likely to occur with an interactive narrative because it is easy to manipulate and is more immersive than a traditional narrative. However, compared with static data visualization, interactive data visualization may increase information avoidance because manipulation of the interactive elements may magnify the difficulty of understanding the data and graphs, thus dominating cognitive resources and resulting in overload. As the effect of interactivity may vary under different modes of presentation, we propose:

#### **Hypothesis 4**

The mode of information presentation moderates the effect of interactivity (vs. non-interactivity) on users’ information avoidance such that the effect of interactivity will more persuasive under the narrative than the data visualization condition.

## Methods

### Participants

The study was in accordance with the 1964 Declaration of Helsinki and its later amendments or comparable ethical standards. Ethics approval was obtained from Zhongnan University of Economics and law Ethics Committee. Information about the study was sent via the WeChat app to undergraduate students at a public university in central China. The screening was performed using a 2-min online survey. Eligible participants were selected according to the following criteria: [1] female, [2] unvaccinated and not due to be vaccinated in the next 3 months, [3] aged 18 to 22, [4] Han nationality, [5] not a medical student and no immediate family members working in medicine, and [6] frequent social media use. This process resulted in the recruitment of 183 participants. A power analysis was conducted using Gpower software [44]. The analysis indicated that a total sample size of 171 was required to achieve medium to large effect sizes (Cohen’s f = 0.25) [45]. Therefore, we randomly selected 180 of the 183 eligible participants. Every participant received monetary compensation after completing the study. All participants gave written informed consent before participation.

### Experimental conditions

**Fig. 1:**
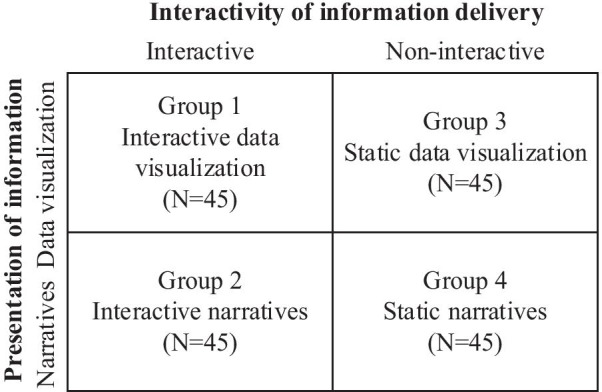
Experimental design (N = 180)

**Fig. 2:**
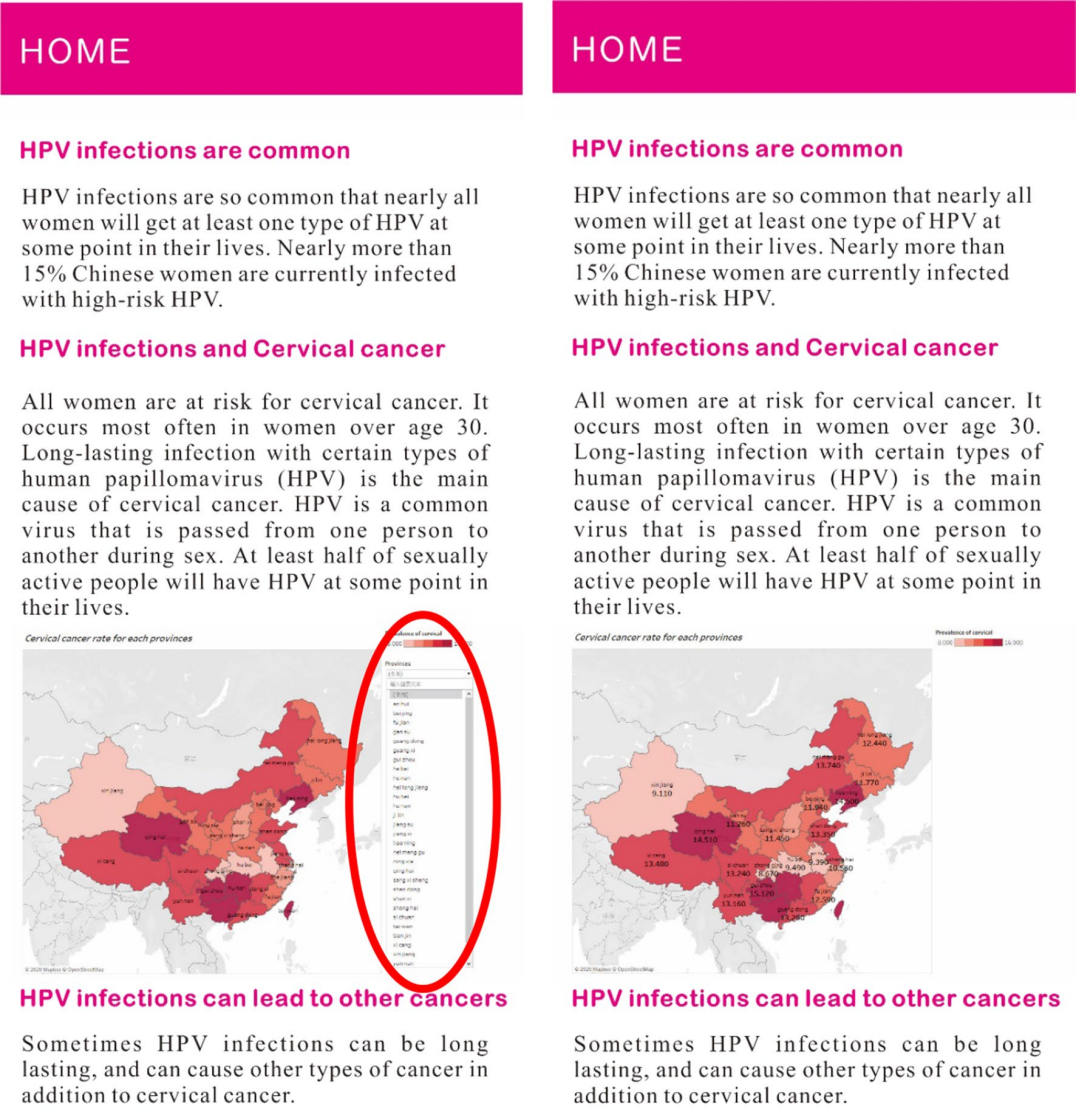
Data visualization with high interactivity (left) and low interactivity (right). *The materials were translated from Chinese and interactive part was circled in red

**Fig. 3:**
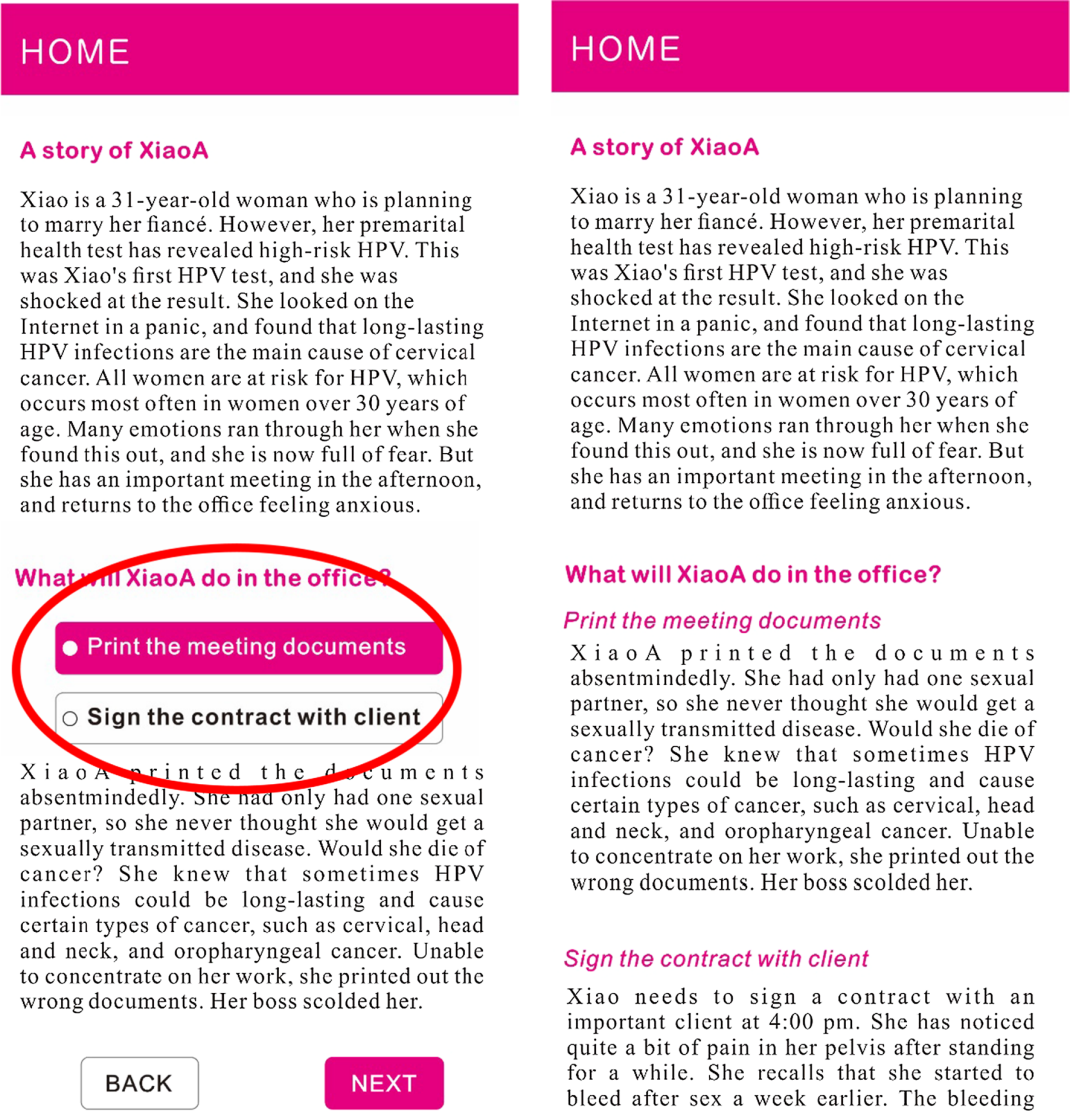
Narratives with high interactivity (left) and low interactivity (right). *The materials were translated from Chinese and interactive part was circled in red

A 2 (interactivity of information delivery: interactive vs. non-interactive) * 2 (mode of information presentation: narrative vs. data visualization) between-groups design was adopted. The stimulus materials were designed based on data from the National Health Commission of the People’s Republic of China and National Bureau of Statistics of China, which provide information on HPV infection in China over the past 18 years, the relationship between HPV infection and different cancers, and the effectiveness of the HPV vaccine in preventing cancer. Using these data, a professional Web designer constructed four Web pages, one for each condition: a. interactive data visualization; b. static visualization; c. interactive narrative; and d. non-interactive narrative. The 2*2 experimental design is shown in Fig. 1. All conditions contained HPV/HPV vaccine knowledge, such as “HPV infections are common,” “HPV infections cause cervical cancer,” “Some HPV infections can lead to other cancers,” and “Prevent cancer with the HPV vaccine.” The data visualization conditions conveyed knowledge via data visualization whereas the narrative conditions communicated knowledge using a short story written in the third person. The interactive and static data visualization conditions shared the same information. The only difference between them was whether interactive features were included. Participants in the interactive condition could change the online content using mouse-based actions such as clicking and dragging. For example, there was a heat map with HPV infection rates for each province in China in 2018. The clickable heat map allowed participants to freely check each province by clicking on the province or selecting the province from a drop-down list. The heat map also contained a mouse hover feature (Please See Fig. 2, left). Participants in the static data visualization condition received the same information but with no interactive elements (Please See Fig. 2, right). The narrative condition included a story about a fictional young female named XiaoA who had an HPV infection. The story contained four sections: being diagnosed with HPV during a premarital examination, working at the office, dealing with her interpersonal relationship, and talking to her doctor. Under the interactive condition, participants were allowed to choose the path XiaoA took by clicking the buttons below each event (Please See Fig. 3, left). In the static condition, a summary text was provided (Please See Fig. 3, right). The information provided in the data visualization was delivered in the narrative via the characters’ (e.g. the doctor’s) dialogue or recall.

### Procedure

The study was conducted in a classroom with a projector. After a brief introduction to the study, participants were asked to sit a certain distance from each other to prevent discussion. Then, they were asked to scan a QR code shown on the projector to enter the experiment’s Web page. On the first page, they read the instructions and answered a set of questions to assess their knowledge of HPV and the HPV vaccine. Then they were randomly assigned to one of four experimental conditions by clicking the link at the end of instruction page. The experiment was presented on participants’ smartphones where they could read or interact with the information. Finally, after viewing the health information, they completed an online questionnaire that measured their information avoidance, intention to obtain the HPV vaccination, and demographic information. The whole process lasted about 40 min.

### Measures

#### Induction checks

The effectiveness of the interactive treatment was assessed with two items adapted from Oh and Sundar [24]. Participants were asked to indicate “how interactive the web page is” and “how the page allows you to perform interactive actions” on a 7-point Likert scale (7 = very much, 1 = not at all). Similarly, the effectiveness of the information presentation manipulation was measured using items adapted from Zhang and Wang [8]. Participants rated whether the information they read was supportive of obtaining the HPV vaccine using (a) narratives, for example, lay-reports or messages containing personal experiences, or (b) data visualization, for example, encouraging vaccination by providing and explaining statistical evidence or graphs (1 = narrative to 7 = data visualization).

#### Behavioral intention

Behavioral intention over the next 3 months was measured using three items adapted from Gerend et al. [46]. The items were “I intend to search for more information related to HPV vaccination,” “I may obtain the HPV vaccination,” and “I intend to consult with friends or doctors about the HPV vaccine” (Cronbach’s α = 0.899). Responses were given on a 7-point Likert scale.

#### Mediating variables

Information avoidance was measured using three items modified from Howell and Shepperd [47]: “I would avoid reading news related to HPV,” “I would rather not read this news,” and “I want to read the news immediately” (reverse-scored item) (Cronbach’s α = 0.782). Responses were given on a 7-point Likert scale.

## Results

### Descriptive statistics and induction check

The participants reported a medium level of knowledge about HPV (M = 4.80, SD = 1.21) and a low level of knowledge about HPV vaccination (M = 3.67, SD = 1.90). We compared the participants’ characteristics across the four treatment groups using ANOVA and Fisher’s exact test. The characteristics of the participants across all four groups were balanced, with no significant differences among the group (Please see Table 1). Also, all of the participants were female undergraduate students with Han nationality. Thus, the results suggested that random assignment was appropriate.

**Table 1:** Participants’ characteristics by treatment group

Variable	Group 1	Group 2	Group 3	Group 4	*P *value
*Age (years)*	19.41	18.78	20.17	19.54	*P* = *0.76*
*Birthplace*					*P* = *0.89*
North	5 (4.4)	4 (8.8)	4 (8.8)	3 (6.7)	
South	6 (13.3)	9 (15.6)	5 (11.1)	8 (11.1)	
East	7 (15.6)	5 (11.1)	8 (17.8)	9 (20)	
West	10 (22.2)	11 (24.4)	7 (15.6)	12 (26.7)	
Central	17 (44.4)	16 (40)	21 (46.7)	13 (35.6)	

The manipulation of interactivity and mode of information presentation was successful. An independent t-test demonstrated that females who viewed the interactive information had significantly higher perceptions of interactivity (M _interactivity_ = 6.48, SD = 0.84) than participants who viewed the noninteractive information (M _non-interactive_ = 1.21, SD = 0.97), P < 0.001). Similarly, participants who were in the narrative condition perceived significantly higher levels of storytelling (M _narratives_ = 1.24, SD = 0.46) than females in the data visualization condition (M _data visualization_ = 6.86, SD = 0.52), P < 0.001. Table 2 summarizes the descriptive statistics of information avoidance, behavioral intention, and knowledge about HPV and HPV vaccination.

**Table 2:** Descriptive statistics of the main variables

Variables	M	SD	Items	N
Information avoidance	2.46	1.34	3	180
Behavioral intention	5.02	1.65	3	180
Knowledge about HPV	4.80	1.21	4	180
Knowledge about HPV vaccination	3.67	1.90	3	180

### Effects of experimental conditions

The interactivity of the information significantly influenced the information avoidance of participants: interactivity (M = 2.71, SD = 1.46) resulted in higher information avoidance than non-interactivity (M = 2.21, SD = 1.27), F [1, 174] = 5.11, *p* < 0.05, η^2^_partial_ = 0.03. A significant interaction effect was not observed on information avoidance.

**Fig. 4:**
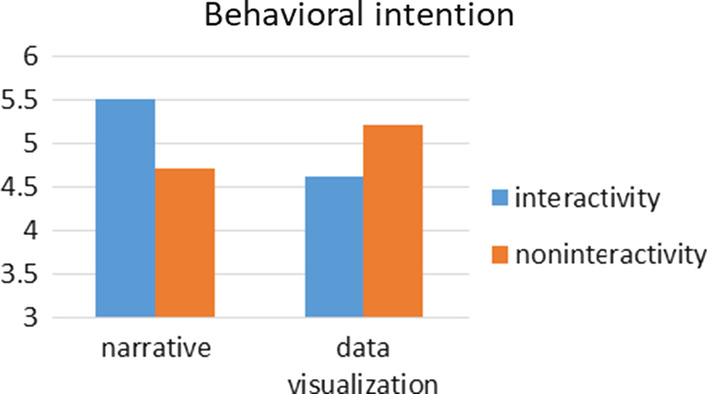
Effects of interactivity on behavioral intention in the narrative and data visualization conditions

Next, we assessed the participants’ behavioral intentions. The presentation of information significantly moderate the effect of interactivity on behavioral intention: F [1, 174] = 13.01, *p* < 0.001, η^2^_partial_ = 0.07. A post-hoc test revealed that under the narrative condition, interactivity (M = 5.51, SD = 1.47) resulted in higher behavioral intention to get the vaccination than non-interactivity (M = 4.72, SD = 1.46). In comparison, under the data visualization condition, non-interactivity (M = 5.21, SD = 1.71) resulted in higher behavioral intention to get the vaccination (M = 4.63, SD = 1.81) than interactivity (In Fig. 4).

### Hypothesis testing and research questions

To answer the research questions and test the hypotheses, we tested a moderated mediation model in which significant mediation effects were assumed to be moderated by the mode of presentation. The model fit showed satisfactory results: χ^2^ = 35.50, df = 20, χ^2^/df = 1.77, RMSEA = 0.06, CFI = 0.99, TLI = 0.97. Interactivity (vs. non-interactivity) increased information avoidance (B = 0.588, SE = 0.183, P < 0.01) (H1) and information avoidance further decreased willingness to receive the HPV vaccination (B =  − 0.25, SE = 0.08, P < 0.01) (H2). H1 and H2 were supported. The indirect effect of interactivity on behavioral intention was significant (indirect effect = -0.14, P < 0.05) and information avoidance played a full mediating role. The finding corroborated H3.

**Fig. 5:**
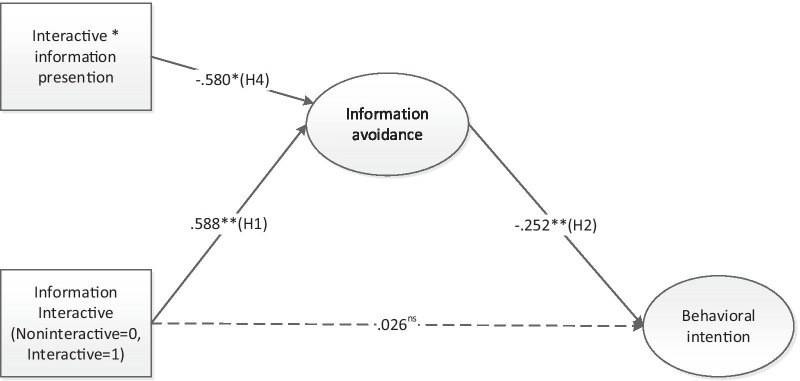
Moderated mediation model. χ^2^ = 35.50, df = 20, χ^2^/df = 1.77, RMSEA = 0.06, CFI = 0.99, TLI = 0.97. *Note*: The statistics represent standardized regression coefficients

According to H4, the indirect relationship between interactivity and behavioral intention through information avoidance is moderated by the mode of presentation. The interaction effect between interactivity and information presentation was significant influence information avoidance (B =  − 0.58, SE = 0.268, P < 0.05). H4 was also corroborated. We then examined the conditional indirect effects of the interactivity on information avoidance at two values of information presentation. Under the narrative condition, the mediation effect was significant: interactivity increased willingness to obtain the injection (B =  − 0.23, SE = 0.10, P < 0.05). However, in the data visualization condition, intentions to obtain the HPV vaccine did not differ significantly between the interactive and non-interactive conditions (Please See Fig. 5).

## Discussion

In this study, a moderated mediation model was used to test the hypothesis of the study, connect Web-based interactivity with HPV information avoidance and willingness to obtain HPV vaccination. We also explored whether using interactivity in the presentation of information increased information avoidance and HPV vaccination intention. The main findings of the study were that Web-based interactive technology may not be easily accepted but **narratives** may improve understanding, acceptability and effectiveness of the technology among female participants. These findings are consistent with those reported previously indicating that participants without major technical challenges have positive perception of interactive tool [48].

We further examined the influence of Web-based interactive technology on information avoidance among young females, which is often ignored. Quantitative analysis revealed that Web-based interactive information might not be readily accepted by young females. Female participants in interactive group reported higher information avoidance (M = 2.71, SD = 1.46) than those who viewed non-interactive information (M = 2.21, SD = 1.27), F [1, 174] = 5.11, *p* < 0.05. The results are consistent with the limited capacity model of motivated mediated message processing suggesting that complicated interactive features compete for limited cognitive resources, leading to cognitive resource overload and information avoidance [28].

Although Web-based interactive technology hinders the acceptability of messages among young females in general, the presentation of interactive information moderates the effect. The **narrative** may help improve understanding, acceptability and effectiveness with Web-based interactive messages. Under the narrative condition, Web-based interactivity significantly decreased information avoidance, whereas under the data visualization condition, interactivity increased information avoidance. When the information was presented via data visualization, the collective aggregated data hindered users from understanding the real meaning of the data. The graphs and numbers emphasizing several HPV-associated cancers and the exponential growth rate of infection, together with the enhanced complexity of manipulating the information, increased information avoidance. Conversely, the interactive narrative presented as a user-friendly Webpage allowed the users to decide the direction of the plot and implicitly conveyed the consequences of HPV and the necessity of HPV vaccination. The findings are consistent with those of previous research. Xu and Sunder [28] also indicated that because interactive data visualization requires a high level of cognitive engagement, users spend less time on highly interactive websites. Interactive narratives, in contrast, facilitate deeper immersion into the story’s characters [42] and reduce information avoidance [10].

Web-based interactive technology did not affect the willingness to accept the HPV vaccine directly but information avoidance played a complete mediatory role. If the manipulation of interactive features demands users’ limited cognitive resources, they may have insufficient resources to understand or concentrate on the content. This would make it difficult for young females with insufficient cognitive resources to engage themselves in the information and hence increase their vaccination intention. Many studies have investigated the mediating effect of message involvement on the effect of interactivity on behavioral intention. Generally, participants engage more in information presented, generate more positive attitudes toward the information, and are more eager to follow the guidance of the information [49]. Considering the results of previous studies, we assume that message involvement only happens when the interactive function matches the information well, or when users have enough resources to handle interactive information. The path from interactivity to behavioral intention may first go through information avoidance, allowing immersion, information involvement, or empathy to happen. Further study on distal mediators is required.

Finally, presentation of information moderated the effect of interactivity on behavioral intention to receive HPV vaccination. The difference between the effect of Web-based interactivity and noninteractivity on vaccination willingness was only significant under the narrative condition. Previous studies on the effect of interactivity have reported inconsistent results. One study found that Web-based interactivity was more effective in recommending health behaviors than noninteractive media [50]. However, another found no significant difference between interactive and noninteractive media [51]. The results of the current study may explain these contrasting results. On one hand, data visualization was associated with lower willingness to receive vaccination among females for both web-based interactive and non-interactive groups. Both conditions increased information avoidance and decreased vaccination intention. Maybe the presentation of collective data, which can easily result in health information avoidance, is not the best approach to persuade females to receive HPV vaccination. On the other hand, interactive narrative increased willingness to receive vaccination among females than static narrative information. Young women find Web-based interactive information compelling or convincing only when they have enough resources to handle interactivity. For example, the interactive narrative effectively convince females to get vaccinated.

## Limitations

The current study has some limitations. First, the number of participants was small and comprised only educated female participants. They mostly have had a higher level of mathematical literacy than females with a lower education level. Thus, they may have been more able to manipulate the interactive information and understand the data visualization, despite the other complicated interactive features competing for their limited cognitive resources. Therefore, this study may not repesent the general young female population, which limits the generalizability of the results. The moderate between-groups effect is likely to be more significant in non-student samples. Second, it would be better to explore participants’ behavioral intentions over a longer-term (e.g., 2 weeks), to provide a more valid assessment of the causal relationship between health message and vaccination intention. Third, the stimulus materials for the interactive narratives was presented in text boxes, which required little more interaction than flipping from page to page. Finally, we used “tableau” software to create the graphs for the interactive data visualization, which were embedded within the web page; however, because the service of “tableau gallery” is based in the USA, the loading speed for the interactive graphs varied based on the speed of the mobile sensor nodes. This meant that the participants’ experiences of the interactive data visualization differed, although they had read the textual information first.

## Conclusions

Although researchers, clinicians and government are developing Web-based interactive tools to provide healthcare information that is specific, acceptable and interactive for among young females [10]. This study provides both theoretical and practical implications of the Web-interactive interface tools. From the theoretical perspective, the established research model provides a new mechanism to explain the persuasive effect of Web-based interactive information on health behavioral intention. The relationship between Web-based interactivity and behavioral intention through information avoidance was moderated by the mode of presentation. The constructs of information avoidance and presentation of information are merged into the model, which effectively helps to understand how and why the Web-based interactivity works, and hence how outcomes can be further improved. From the practical perspective, this research is particularly important for public health department and mass media as it provides insights on females, the target of HPV vaccination. First, the level of interactivity is not the higher the better. Higher interactivity may lead techno-anxiety and it should have been taken into consideration. Young females tend to avoid HPV-related information because they fear manipulation by complex information. Second, simple and familiar designs of interactivity are preferred by females for receiving health information, and these formats decrease their information avoidance. Young females are more likely to be persuaded by an interactive narrative than by interactivity in statistical or graphical information. An appropriate plot of narratives can decrease information avoidance, increase sympathy towards the patient portrayed in the story, and hence vaccination intention. Because computer literacy appeared to be essential for information processing, computer skills should be taken into account when designing interactive health information.

## Data Availability

The datasets used and/or analyzed during the current study are available from the corresponding author on reasonable request.
